# Computational and structure-guided design of phosphoinositide substrate specificity into the tyrosine specific LMW-PTP enzyme

**DOI:** 10.1371/journal.pone.0235133

**Published:** 2020-06-25

**Authors:** Eyong Egbe, Colin W. Levy, Lydia Tabernero

**Affiliations:** School of Biological Sciences, Faculty of Biology Medicine and Health, University of Manchester, Manchester Academic Health Science Centre, Manchester, England, United Kingdom; Universite Paris Diderot, FRANCE

## Abstract

We have used a combination of computational and structure-based redesign of the low molecular weight protein tyrosine phosphatase, LMW-PTP, to create new activity towards phosphoinositide substrates for which the wild-type enzyme had little or no activity. The redesigned enzymes retain catalytic activity despite residue alterations in the active site, and kinetic experiments confirmed specificity for up to four phosphoinositide substrates. Changes in the shape and overall volume of the active site where critical to facilitate access of the new substrates for catalysis. The kinetics data suggest that both the position and the combination of amino acid mutations are important for specificity towards the phosphoinositide substrates. The introduction of basic residues proved essential to establish new interactions with the multiple phosphate groups in the inositol head, thus promoting catalytically productive complexes. The crystallographic structures of the top-ranking designs confirmed the computational predictions and showed that residue substitutions do not alter the overall folding of the phosphatase or the conformation of the active site P-loop. The engineered LMW-PTP mutants with new activities can be useful reagents in investigating cell signalling pathways and offer the potential for therapeutic applications.

## Introduction

Phosphorylation and dephosphorylation events are critical to regulate physiological processes in eukaryotic cells. There are 125 Protein Tyrosine Phosphatases (PTPs) in the human genome, divided into cysteine-based, aspartate based, and histidine-based PTPs depending on the nucleophile [1]. Cys-based PTPs have a structurally conserved phosphatase domain, and catalytic C(X)_5_R motif in their active site (P-loop), where the Cys is the nucleophile and the Arg binds to the phosphate group in the substrate. A conserved aspartic acid, generally found on a loop close to the active site (WPD-loop or D-loop, in case of LMWPTP), acts as the general acid [2, 3]. Although Cys-based PTPs exhibit the same mechanism of catalysis, the substrate specificity of this group is very diverse, with substrates ranging from Tyr, Ser, and Thr-phosphorylated proteins to phosphoinositides [1]. The difference in the shape of the active site and the residues at the binding interface determine the diversity in substrate specificity. Consistent with this, phosphoinositide phosphatases, PTEN and Myotubularins, possess a deep, wide active site that serves to accommodate its bulky physiological substrates [4, 5], whereas Tyr-specific PTPs have a narrow and deep active site [6].

Deregulation of phosphoinositide metabolism occurs in various diseases. Malfunction of Myotubularins (MTM) and Myotubularin-related proteins (MTMR), which dephosphorylate the D3 phosphate PI[3]P and PI[3,5]P2, is responsible for a number of myopathies [7]. Mutations in MTM1 cause X-linked Myotubular myopathy (XLMTM), a fatal congenital muscle disease [8]. Malfunction in MTMR2 leads to Charcot-Marie-Tooth Disease type 4B (CMT4B) [9]. Mutations in FIG4, which cleaves the D5 phosphate of PI[3,^5^]P2, leads to CMT4J [10], Amyotrophic Lateral Sclerosis (ALS) [11] and Yanus Varon syndrome [12]. Loss of Sac1-domain phosphatase Synaptojanin 1 is associated with early onset Parkinsonism and epilepsy [13, 14].

Protein engineering has emerged as a key tool in biotherapeutics. In particular, recent examples of engineered forms of PTPs have been used to improve target delivery to specific cellular compartments [15], as replacement therapy to improve muscle weakness in Myotubularin knock out mice with symptoms of XLMTM [16], or to increase tumour suppressor activity of PTEN [17, 18]. Development of a biotherapeutic pipeline is complex and expensive. Consequently, the ability to modify the substrate specificity of a single PTP enzyme in order to target lack of function mutations of genes involved in disease pathways offers an opportunity for enzyme replacement therapy applications. This will significantly reduce production cost and make this research domain more attractive for investment.

Engineered PTPs could also serve as valuable tools to study phosphorylation-dependent pathways and their modulation in different model organisms. Previous examples were reported for the heterologous expression of human phosphatases used to reconstitute the mammalian PI3K/PTEN/Akt pathway and study the regulation of cell growth in *S*. *cerevisiae* [19]. In another study, replacement of essential yeast genes with their corresponding human orthologues showed that over 200 yeast essential gene functions were complemented by the human genes [20].

The current availability of over 300 PTP crystal structures and complexes with 178 ligands provides an excellent resource to establish the key structural determinants of substrate specificity. This information offers a robust platform for engineering new functions or substrate affinities into these enzymes. In this study, we explore the feasibility of designing new substrate specificity into the Low Molecular Weight Protein Tyrosine Phosphatase (LMW-PTP), the smallest member of the PTP family. LMW-PTP is a well-characterised phospho-tyrosine specific PTP, widely expressed in human tissues [21, 22] and conserved through evolution. Our approach involved a combination of rational and computational enzyme design, exploiting existing structural knowledge of the template and target enzymes and the Rosetta Macromolecular Modelling suite [23]. The *RosettaEnzyme* application is one of the most commonly used computational design tool used for enzyme design [24], and proved successful in a number of enzyme design examples [25], thus justifying our choice.

The redesigned enzymes were subsequently validated experimentally. We demonstrate here that minimal re-design of LMW-PTP, by mutating only two residues, is sufficient to introduce new enzymatic activity towards four different phosphoinositide (PI) substrates. Crystallographic analysis confirmed that the mutations do not alter the three-dimensional structure of LMW-PTP, and that they are compatible with binding to PI[3,5]P_2_ substrate.

This is the first reported example of successful engineering new substrate specificity into a PTP and offers unexplored opportunities for further development. The ability to generate a toolbox of small phosphatases with *tuneable* substrate specificity would enable to select different substrate binding capabilities with a wide range of potential applications as research tools and therapeutics.

## Methods

### Sequence and structural alignments

The 3D structures of template enzyme LMW-PTP, 1XWW [26] and target phosphoinositide phosphatase myotubularin MTMR2, 1ZVR [4], were obtained from the Protein Data Bank (PDB) and their active sites were aligned using CCP4 Superpose [27]. The aligned region (Lys6 to Lys28) corresponds to the β-strand before P-loop, the P-loop, and 3 turns of the α-helix after the P-loop, and it was chosen because it is structurally conserved in phosphatases. Structural analyses were done using PyMOL (MacPyMOL: PyMOL v1.8.0.3 Enhanced for Mac OS X (Schrödinger LLC)). Sequence alignments of LMW-PTP and phosphoinositide phosphatases were done using Clustal Omega [28] and visualised with Jalview [29]

### Receptor and ligand preparation

The template model, 1XWW was relaxed using the *Rosetta_relax* application using all-heavy atom constrain short protocol [30] and the structure with the lowest energy score was selected as receptor for enzyme design. The coordinates for PI[3,5]P_2_, were extracted from the MTMR2 crystal structure, PDB ID:1ZVR [4] and used as ligand in the design and docking calculations. Experimental evidence suggests the inositol head group is the critical determinant of enzyme-substrate interaction [4]. To facilitate the Rosetta simulation process, the poly-carbon tail of the glycerol moiety was deleted, reducing the ligand into a phosphorylated inositol head containing an ethyl group on the D1 phosphate. This reduces the effect of the poly-carbon tail flexibility during design and docking, while maintaining the charges present in the physiological ligand [31]. Ligand conformers are needed to model ligand flexibility during design and docking and to obtain these, molecular topologies of the ligands were generated using PRODRG server [32], and partial charges and geometry optimised using Avogadro molecular editor [33]. The resulting optimised ligands were used in Balloon 3D conformer generator [34] to generate ligand conformers. The parameter file (params_file) was created using Rosetta molfile_to_params.py script.

### Rosetta computational enzyme design

The *RosettaEnzyme* design method [24, 35] was used with some modifications to suit the needs of the current research problem. Distance constraints (cstfile) were defined between LMW-PTP WT catalytic residue C12 and the D3 phosphorous atom of the ligand, and between the backbone amide groups at the P-loop (residues G14, N15, C17, R18) and D3 oxygen atoms. Distances from corresponding interactions between PI[3,5]P_2_ and MTMR2 in the crystals structure [4] were used as a reference to define the constraints. Distance constraints were set at 3.8 to 4.2 Å between the backbone amide-nitrogen atoms and D3 phosphorus atom, and 3.2 Å between C12 sulphur atom to D3 phosphorus atom. A residue file (resfile) was created containing the selected residues at the binding interface to be mutated based on their proximity to the P-loop, non-deleterious effect of enzyme activity upon its mutation and suitability to accommodate new interactions. This included L13, I16, W49, N50, Y131, and Y132. For each computational design run, 2000 structures were requested, and the best 20 scoring models were selected amongst the top 100 after eliminating those with distortions in the 3D structure. All Rosetta applications were obtained from: https://www.rosettacommons.org. The volumes of the active sites were measured using the PockDrug-Server [36]. All protein structure images were created with MacPyMOL: PyMOL v1.8.0.3 Enhanced for Mac OS X (Schrödinger LLC).

### Expression and purification of recombinant protein

The coding sequences of human LMW-PTPB (Addgene) and MTMR2 (a kind gift from Prof. M. Clague, University of Liverpool, UK) were cloned into a pNIC28a-Bsa4 vector (Addgene) to generate an N-terminal 6xHis-tagged expression. Site-directed mutagenesis with appropriate primers was performed using Accuzyme DNA polymerase (NEB, UK) and mutations were confirmed by DNA sequencing. Recombinant proteins (LMW-PTP WT, mutants, MTMR2) were expressed in BL21-DE3 *Escherichia coli* cells using 0.1mM Isopropyl β-D-1-thiogalactopyranoside (IPTG) induction overnight at 20°C. Cells were lysed in 50mM TRIS-HCl, 150mM NaCl, 0.1% Triton X-100 (v/v), 10 mM Imidazole pH 7.5 by French press and the supernatant was clarified by centrifugation at 12,500 x g for 1 h 30 min. Enzymes were purified by affinity chromatography using nickel column (GE Healthcare) pre-equilibrated in binding buffer (50mM TRIS-HCl, 500mM NaCl, 20mM Imidazole, pH 7.5) and eluted with 50mM TRIS-HCl, 500mM NaCl, 500mM Imidazole, pH 7.5. This was followed by size exclusion chromatography using Superdex SX-75 column (Amersham) with 50mM TRIS-HCl, 250mM NaCl, 5mM EDTA, pH 7.5 as elution buffer.

### Crystallisation and structure determination

Prior to crystallogenesis LMW-PTP mutant proteins were prepared by removal of their 6His tag with TEV (tobacco etch virus) protease. The cleaved protein was loaded on to a nickel affinity column and the flow through collected. Samples were subsequently further purified using size exclusion chromatography (Superdex SX-75, 50mM HEPES, 250mM NaCl, pH 7). Crystal trials were performed via sitting drop vapour diffusion by mixing equal volumes of protein and reservoir solution (200nl) and incubating at 4°C for 24 hours. The single mutant I16K (9.70 mg/mL) was crystallised in 0.2M Sodium acetate trihydrate, 0.1M Bis-Tris, 25% PEG3350, pH 6.5 whilst the double mutant L13R-I16K (10.5 mg/mL) was crystallised in 0.1M Bis-Tris, 25% PEG3350, pH 5.5. Crystals were cryo-protected (15% PEG200 in mother liquor) prior to flash cooling in liquid nitrogen. Complete diffraction data, extending to 1.8Å and 2.0Å for the single and double mutant forms respectively, were collected on i04 at Diamond Light Source (DLS). All data were subsequently scaled and merged in Xia2 [37], data collection and refinement statistics are presented in Table 3. Initial phases were determined by molecular replacement in Phaser [38] with a search model derived from structure 1XWW. Model building, refinement and validation were performed using COOT [39], Phenix [40] and Molprobity [41]. PDB-REDO [42] was integrated into the iterative rebuild and refinement process.

### Phosphatase activity measurement

Phosphatase activity assays were done using the pNPP substrate. The reaction mixture contained 5 μg of enzyme and 1 mM substrate in a 150 μl reaction mixture (with 50 mM TRIS, 50 mM Bis-Tris, 100 mM Sodium acetate, pH 5.5 as assay buffer) and incubated for 30 min. at 37 °C. The testing of mutants towards phosphoinositides and kinetics characterisation were done using malachite green assay (SIGMA). For this, 5 μg of enzyme was added to 150 μM substrates (diC8 phosphoinositides from Echelon Bioscience, UT, USA) in a 50 μl reaction mixture composed of 50 mM TRIS, 50 mM Bis-Tris, 100 mM Sodium acetate, pH 5.5 for LMW-PTP-mutants and pH 7.5 for MTMR2. The reaction was incubated for 45 min. at 37 °C. For kinetic parameters determination, 0.5–5 μg of mutant was added to 0–150 μM diC8 phosphoinositides (with exception to PI(3)P, 0–250 μM) and incubated for 30 min. at 37 °C (Note: for some substrates it was not possible to reach saturation within the concentration range compatible with the malachite assay, thus kinetic calculations were not carried on). The reaction was quenched by adding 15 μl malachite green reagent and further incubated for 30 min. to allow for colour development. The absorbance was measured at 620 nm on a plate reader. The amount of free phosphate released was interpolated from a phosphate standard curve generated using 50–3000 pmoles of phosphate standard to calculate initial velocities. Kinetic parameters were calculated from a nonlinear regression curve fitting analysis using the Michaelis-Menten equation in GraphPad Prism 7.0. The results are represented as the mean± SEM (standard error of the mean) of two representative experiments consisting of triplicates of each data point.

## Results

### Structural comparison of LMW-PTP and phosphoinositide phosphatases

Substrate specificity in PTPs is defined by both the shape and amino acid composition of the active site and the conserved phosphate-binding loop (P-loop). In particular, the depth of the active site discriminates binding to phospho-tyrosine (pTyr) versus the shorter phospho-serine/threonine (pSer/pThr) substrates. Thus pTyr-specific PTPs have a narrow and deep active site (about ~ 9Å in depth) [6, 43], whereas dual-specificity PTPs have a shallower active site. In the case of phosphoinositide (PI) phosphatases, such as phosphatase and tensin homologue (PTEN) and myotubularins, the active site is wider and rich in basic residues required for binding to the several phosphate groups in the substrate [4, 5].

**Fig 1:**
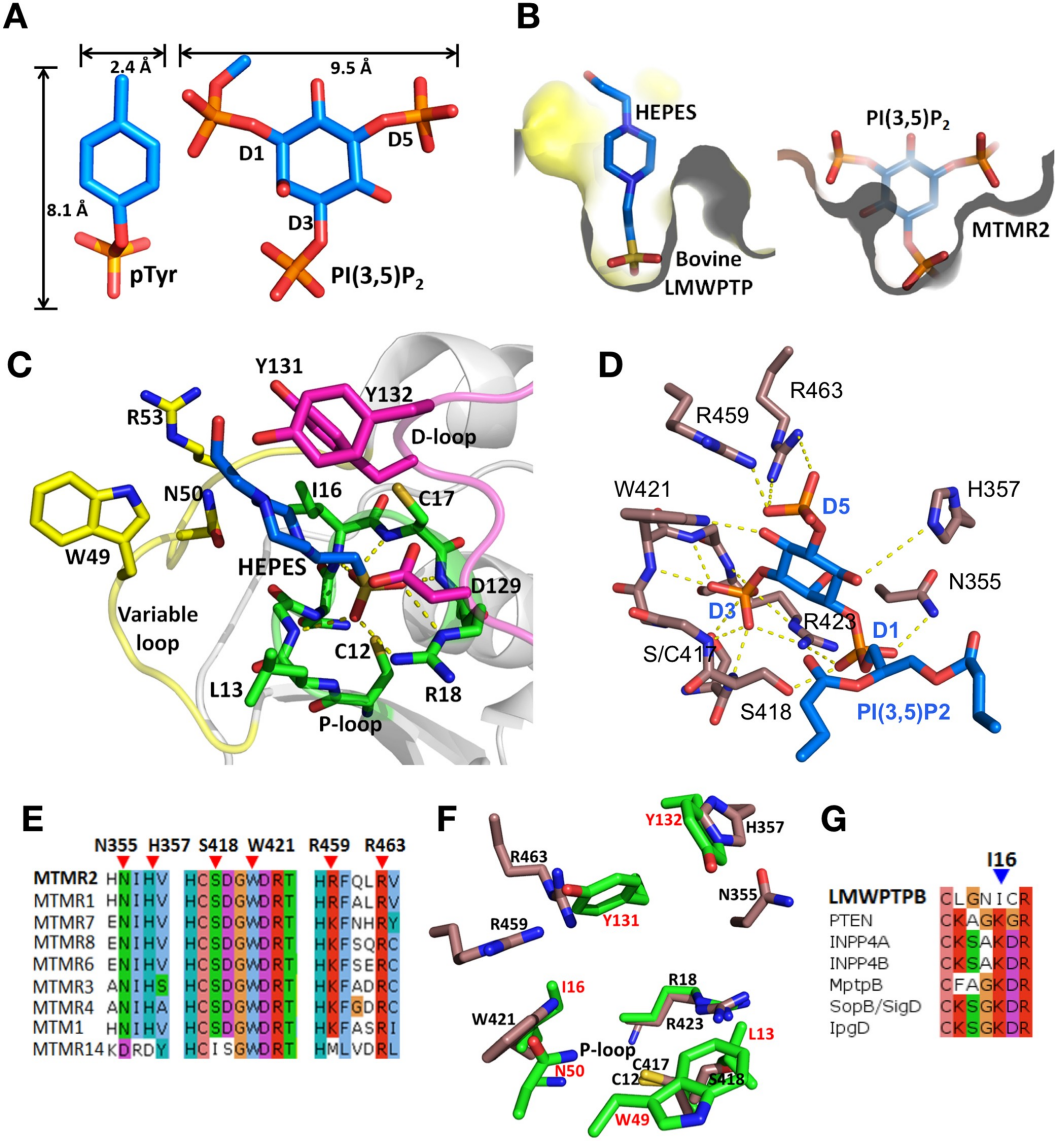
Structural comparison of LMW-PTPB and phosphoinositide phosphatases. **A**) Dimensions of the substrates of LMW-PTP (pTyr) and Myotubularin (PI[3,5]P2). **B**) Cross section of the active site of Bovine LMW-PTP (left) in complex with HEPES (4-(2-HydroxyEthyl)-1-Piperazine EthaneSulfonic acid) (PDB ID: 1DG9 [43]) and MTMR2 (right) in complex with PI[3,5]P2 (PDB ID: 1ZVR [4]). **C**) Cartoon and stick representation of LMW-PTP in complex with HEPES (blue sticks). The main regions of the active site include the P-loop (green), D-loop (purple) and Variable loop (yellow). Side chains of residues in the binding interface that are in close proximity to the ligand are shown as sticks. Hydrogen bonds (yellow dotted lines) between the ligand and active site pocket residues are labelled. **D**) Stick representation of MTMR2 (brown) in complex with PI[3,5]P2 (blue), showing interactions (yellow dotted lines) between the ligand and the protein. The catalytic Cys417 is mutated in the structure to a Ser (PDB ID: 1ZVR). **E**) Sequence alignment of active members of the myotubularin family shows the conservation of residues that interact with the ligand. The reference for numbering is MTMR2. The red triangles indicate conserved residues that interact with the ligand atoms other than in D3 phosphate (interactions with D3 phosphate is a common feature amongst PTPs). **F**) Superimposition of the active sites of LMW-PTP and MTMR2 showing selected target residues for design (green sticks and red text) and key substrate interacting residues (brown sticks) respectively. **G**) Sequence alignment of the P-loop of LMW-PTP and phosphoinositide phosphatases in mammals (PTEN, INPP4A, INPP4B) and bacteria (MptpB, SopB/SigD, IpgD). The blue triangle indicates a conserved lysine that is important for phosphoinositide binding.

To establish the pre-requisites for redesign we compared the architecture of the active site of LMW-PTP to that of myotubularin-related protein 2 (MTMR2) and PTEN, as well as the size of their respective phospho-substrates. The physiological substrates of LMW-PTP are pTyr proteins. In terms of dimensions, pTyr is similar in depth to the PI head (Fig 1A). However, the cross-sectional width of the PI head of PI[3,5]P_2_, for example, is four times wider than pTyr, due to extra phosphate groups (D1 and D5) extending from the cyclohexane ring. These differences suggest that a wider pocket, similar to that found in myotubularin, may be required in LMW-PTP in order to accommodate PI ligands (Fig 1B).

The ligand-binding interface of LMW-PTP comprises the active site P-loop, the adjacent DPYY-loop (D-loop) and the variable region or variable loop (Fig 1C). The catalytic residues C12 (nucleophile) and R18 (phosphate binding) are located in the P-loop, and D129 (general acid/base) in the D-loop. The Y131 and Y132 residues in the D-loop and W49 in the variable region form a high wall at the opening of the active site cleft that deepens the active site pocket in LMW-PTP rendering it incompatible with binding of a PI ligand (Fig 1B).

Previous studies emphasised the importance of charge-charge interactions between basic residues in lipid phosphatases and their PI substrates. In the crystal structure of MTMR2 in complex with PI[3,5]P_2_ [4] the side-chains of R423, R459 and R463 form charge-charge interactions with the D3 and D5 phosphates (Fig 1D). These three Arg residues are highly conserved in the myotubularin family (Fig 1E) and are essential for catalysis and substrate specificity [44]. Mutagenesis studies in PTEN also demonstrated the importance of another three conserved basic residues (K125, K128, and H93) for specificity towards PI[3,4,5]P_3_ [5].

Whereas the substrate-binding interfaces of PI phosphatases are enriched with basic residues, the binding interface of LMW-PTP has only two basic residues, the catalytic R18, and R53 in the variable region (Fig 1C). In the structure in complex with HEPES (4-(2-HydroxyEthyl)-1-Piperazine EthaneSulfonic acid) [43], R18 interacts with the sulfonic group (Fig 1C) in a similar manner to R423 in MTMR2 binds to the D3 phosphate in PI[3,5]P_2_, but there are no analogues to R459 or R463 (Fig 1F). The deficiency in basic residues at the interface will need to be taken into account in order to redesign LMW-PTP to bind PIs.

### Enzyme design strategy

For the computational redesign of the LMW-PTP we employed a two-prong strategy. Design strategy 1 (DS1): using *RosettaDesign* with the LMW-PTP wild type structure (LMW-PTP-WT) as a template, and design strategy 2 (DS2): using structure-based rational design to introduce new residues in the template prior to *RosettaDesign*.

In both cases, distance constraints were defined between D3 in the ligand PI[3,5]P_2_ and residues at the P-loop to ensure that it remained bound to the active site during the *RosettaDesign* process. Additionally, we eliminated glycine, proline and cysteine as alternative amino acids in the mutations to avoid unwanted structural alterations, and the catalytic residues (C12, R18, D129), as well as C17, which has a role in protecting C12 from oxidation [45]. Thus the residues selected for mutation (in and around the active site) were L13, I16, W49, N50, Y131, and Y132. Superposition of the active sites of LMW-PTP and MTMR2 shows that the selected residues overlap or are in close proximity with ligand binding residues in MTMR2, confirming their potential for redesign (Fig 1F).

In DS2 we incorporated pre-existing knowledge of interactions between PIs and target phosphatases to design a specific substitution in the LMW-PTP-WT structure, and then used this as the starting template for *RosettaDesign*. Mammalian lipid phosphatases (PTEN, INPP4A, and INPP4B) have a conserved lysine in the P-loop that is important for substrate binding. This lysine is also conserved in bacterial lipid phosphatases such MptpB, SopB/SigD and IpgD [46] (Fig 1G). In the LMW-PTP-WT this lysine corresponds to an isoleucine in position 16 of and thus we used the LMW-PTP-I16K mutant as starting template for *RosettaDesign* in DS2.

### Analysis of the most frequent mutations from the enzyme designs

**Fig 2:**
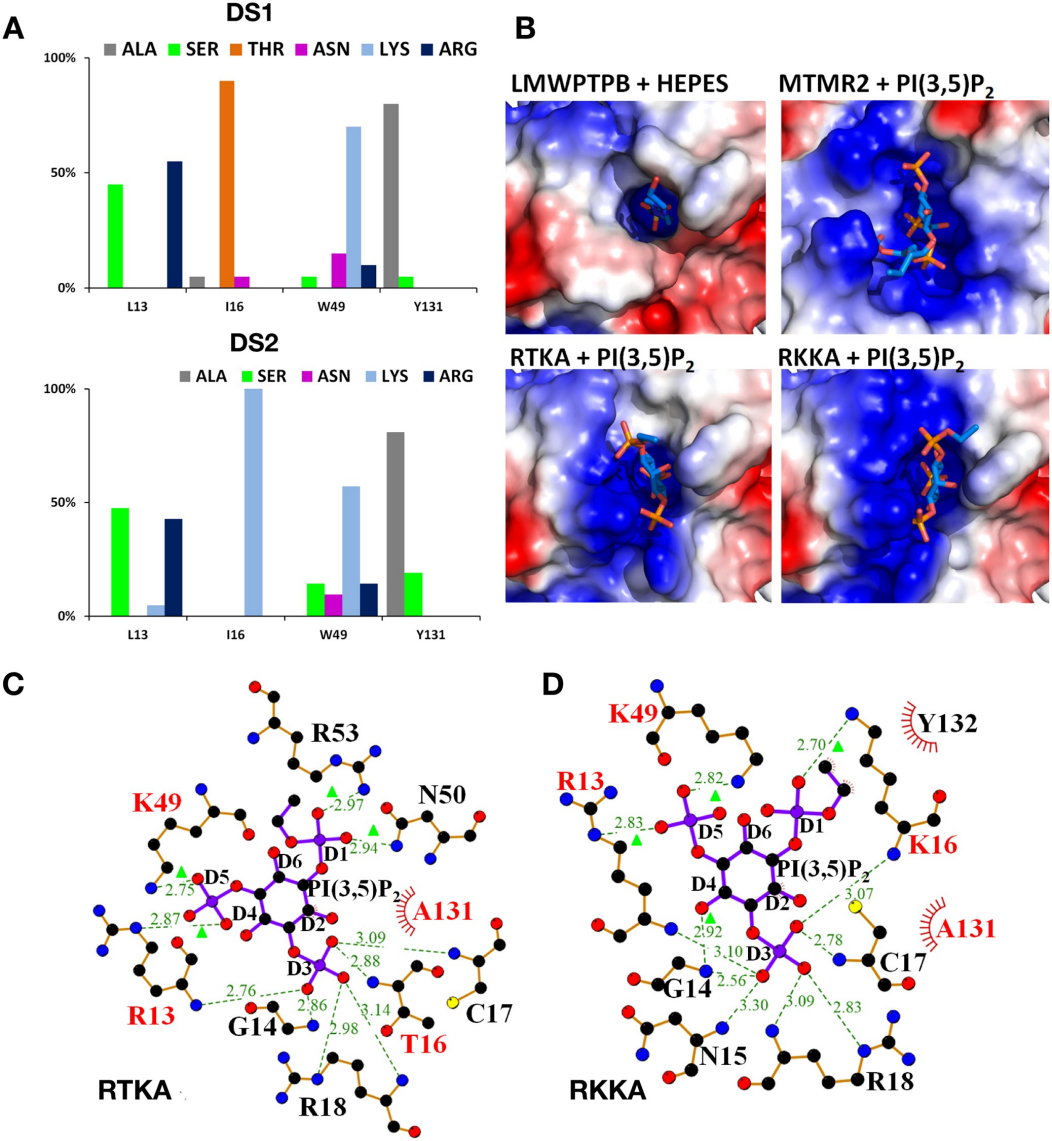
Analysis of mutations introduced during enzyme redesign. **A**) Analysis of the rate of mutation for each strategy. Design strategy 1 with LMW-PTP-WT as model (top) and 2 with LMW-PTPB-I16K as model (bottom) and PI[3,5]P_2_ as ligand. The rate of mutation (Y-axis) at each residue position (X-axis) is calculated using the top 20 Rosetta designs. **B**) The electrostatic surface potential of LMW-PTPB-WT in complex with HEPES (top left), and MTMR2 (top right), RTKA (bottom left), and RKKA (bottom right) in complex with PI[3,5]P2. Interactions calculated with LigPlot [47] between the ligand PI[3,5]P_2_ and the top two scoring Rosetta designs, DE1948 (RTKA)(**C**) and DE316 (RKKA) (**D**). In addition to the interaction between the P-loop residues and D3 phosphate oxygen atoms, new interactions are made with the D1 and D5 oxygen atoms (green triangles) by both newly introduced residues (R13, I16, K49 and K16) and existing WT residues (N50 and R53). Red text represents mutated residues, green dotted lines and green text represent interactions and distances respectively.

Rosetta generated 2,000 protein designs for each input model (LMW-PTP-WT and LMW-PTP-I16K). From the top 100 designs we discarded those containing structural alterations (i.e. P-loop displacements of > 0.5Å between Cα), and then selected the top 20 scoring designs (S1 and S2 Tables) from each strategy (DS1, DS2) to analyse the frequency and type of mutations. The analysis revealed common features in both design strategies. The most frequently mutated residues were L13, I16, W49 and Y131 (> 85% rate of mutation), whereas N50 and Y132 were hardly mutated (<5% rate of mutation). For DS1 (LMW-PTP-WT as template), the most frequently introduced mutations were Arg and Ser at position 13 (L13R or L13S), Thr at position 16 (I16T), Lys at position 49 (W49K) and Ala at position 131 (Y131A). Similarly, for DS2 (LMW-PTP-I16K as template), the most frequently introduced mutations are also Arg and Ser at position 13, Lys at position 49 and Ala at position 131 (Fig 2A).

In both design strategies, the mutation to Arg at position 13 is present in the top 3 designs, and together the high frequency of Lys at position 49, is consistent with the need for additional basic residues to interact with the extra phosphate groups in the ligand PI[3,5]P_2_. The change of Tyr to Ala at position 131 reflects potential clashes of the ligand with the “high wall” at the entrance of the active site formed by Y131 and Y132.

### Enzyme designs show improved binding for PI[3,5]P_2_

The Rosetta binding scores for the PI[3,5]P_2_ ligand to the DS1 top 20 designs, with an average of -9.1 REU (Rosetta Energy Unit), are slightly greater than for the DS2 top 20 designs, with an average of -7.7 REU. This is in clear contrast with the very low values obtained for the control (WT without design), with an average of 4.8 REU for the top 20 structures. For DS1, the best enzyme design DE1948 (with mutations L13R-I16T-W49K-Y131A, hereinafter referred to as RTKA) showed the best binding affinity for PI[3,5]P_2_ with a REU of -10.14 (S1 Table). For DS2, the design DE316 (L13R-I16K-W49K-Y131A or RKKA) showed the best binding affinity for PI[3,5]P_2_ with a REU of -9.17 (S2 Table). In both cases, *RosettaDesign* introduced at least two additional basic residues, which clearly improve the binding affinity for the ligand, compared to the WT enzyme.

Next, we evaluated the impact of the designed mutations on the size of the active site pocket. The volume of the active site of the RTKA and RKKA designs, was 771 and 685 Å^3^ respectively, as measured with PockDrug [36]. This represents an increase in volume of 41% and 25% with respect to the LMW-PTP-WT (pocket volume of 546 Å^3^). This increase in size is sufficient to accommodate the PI[3,5]P_2_ ligand and enables the D3 phosphate to reach the P-loop and catalytic residues.

The introduction of extra basic residues changes the surface potential of the active site pocket into more positively charged, resembling that of MTMR2 (Fig 2B), thus more suitable to accommodate the PI[3,5]P_2_ ligand with three phosphate groups. To understand the role of the newly introduced mutations, we analysed potential ligand interactions between PI[3,5]P_2_ and each top scoring design using Ligplot [47]. For RTKA, the D3 phosphate in the ligand is held by six hydrogen bonds formed with the P-loop backbone amides and the guanidinium side chain of R18 (Fig 2C). These interactions are similar to those observed between LMW-PTP-WT and the HEPES sulphate group (Fig 1C). New interactions are formed between the redesigned residues, R13 and K49, and the D5-phosphate oxygens of PI[3,5]P_2_, as well as between N50 and R53 and the D1-phosphate oxygen. K49, N50 and R53 are located in the loop that forms the variable region (Fig 1C). Similarly, in RKKA the D3-phosphate is anchored at the P-loop by hydrogen bonds with the amide nitrogen atoms, and the new residues R13, K16 and K49 interact with the D1 and D5 phosphate of the ligand (Fig 2D). These interactions are similar to those reported between PI[3,5]P_2_ and MTMR2 (Fig 1D).

In summary, changes in the size and amino acid properties of the active site in the new designs result in novel interactions that favour binding of PI[3,5]P_2_.

### LMW-PTP new designs retain phosphatase activity and show new activity towards phosphoinositides

In order to validate the new designs and to understand the impact of each mutated residue on enzyme activity and specificity, we cloned, expressed and purified 11 different LMW-PTP mutants. These included four single mutants (L13R, I16K, W49K and Y131A), five double mutants (L13R-I16T, L13R-I16K, L13R-W49K, I16T-W49K and I16K-W49K) and two triple mutants (RTK and RKK) (Table 1), harbouring combinations of the mutations identified with *RosettaDesign*. The quadruple mutants (RTKA, RKKA) were not generated because the Y131A mutation results in a dead enzyme (<3% activity respect to WT), possibly due to displacement of the D-loop and shifting the catalytic Asp129 away from the active site.

**Table 1:** Specific activity (nmol/min/mg) of redesigned enzymes towards pNPP and phosphoinositides. All values are means of two independent experiments with triplicates ± SEM, n.a: negligible or no detectable activity. *Mutant enzymes that were selected for kinetic studies.

Enzymes	pNPP	PI(3)P	PI(4)P	PI(5)P	PI(3,4)P_2_	PI(3,5)P_2_	PI(4,5)P_2_	PI(3,4,5)P_3_
**WT**	1303 ± 6	0.53 ± 0.06	1.12 ± 0.01	n.a	n.a	n.a	n.a	n.a
**L13R**	1215 ± 17	4.43 ± 0.21	2.16 ± 0.12	4.44 ± 0.13	n.a	0.33 ± 0.01	n.a	n.a
**I16K**	1711 ± 25	4.66 ± 0.16	5.04 ± 0.17	1.5 ± 0.08	0.05 ± 0	0.65 ± 0.01	n.a	n.a
**W49K**	1639 ± 23	2.67 ± 0.04	n.a	n.a	n.a	0.33 ± 0.02	n.a	n.a
L13R-I16T (RT*)	1423 ± 20	4.43 ± 0.09	3.82 ± 0.26	17.96 ± 0.76	n.a	2.5 ± 0.09	n.a	n.a
L13R-I16K (RK*)	940 ± 26	11.99 ± 0.41	15.79 ± 0.46	42.48 ± 3.11	n.a	12.77 ± 0.67	n.a	n.a
**L13R-W49K**	1385 ± 24	2.94 ± 0.03	n.a	5.66 ± 0.17	n.a	n.a	n.a	n.a
**I16K-W49K**	1408 ± 18	3.77 ± 0.19	1.02 ± 0.05	2.41 ± 0.19	n.a	0.6 ± 0.05	n.a	n.a
**I16T-W49K**	1415 ± 16	0.48 ± 0.03	5.15 ± 0.07	1.75 ± 0.06	0.04 ± 0	2.48 ± 0.14	1.24 ± 0.04	n.a
L13R-I16T-W49K (RTK*)	1360 ± 20	2.02 ± 0.12	3.18 ± 0.24	11.87 ± 0.24	n.a	2.14 ± 0.2	n.a	n.a
L13R-I16K-W49K (RKK*)	858 ± 27	2.37 ± 0.19	3.83 ± 0.17	14.21 ± 0.6	n.a	16.94 ± 1.02	n.a	n.a

Mutant proteins were tested for phosphatase activity towards pNPP and phosphoinositide substrates. All mutants (except Y131A) showed activity towards pNPP comparable to the WT (Table 1), confirming that the mutations do not affect the integrity of the protein or its active site. All mutants were also able to dephosphorylate PI3P, and most of them could dephosphorylate PI4P, PI5P and PI[3,5]P_2_. Exceptions were W49K that showed no activity towards PI4P, or PI5P, and L13R-W49K that did not dephosphorylate PI4P or PI[3,5]P_2_. None of the mutants showed activity towards PI[3,4]P_2_ or PI[3,4,5]P_3_. The WT enzyme showed only very low activity towards PI3P and PI4P. Consequently, four mutants were identified as the best performing mutants with higher activity towards the target substrates: L13R-I16T (RT), L13R-I16K (RK), L13R-I16T-W49K (RTK) and L13R-I16K-W49K (RKK), and were selected for further kinetics studies.

Enzyme kinetics experiments for the dephosphorylation of phosphoinositides reveal that the RT, RTK and RKK mutant enzymes show selective specificity for PI5P, whereas RK has a broader specificity, with activity towards PI3P, PI4P, and PI5P and PI[3,5]P_2_, with the highest efficacy towards PI[3,5]P_2_ (Table 2). Interestingly, the W49K mutation is not an absolute requirement for activity towards PIs, as it appears to have a detrimental effect in L13R-W49K, with only moderate activity towards PI3P and PI5P (Table 1). In fact, the presence of a lysine at position 49 in RTK abolishes activity towards PI[3,5]P_2_ and in RKK lowers its affinity by ten-fold (K_m_ 200 μM). In contrast, the RK mutant, with Trp at position 49 can dephosphorylate four out of the seven PIs tested (Table 2). This suggests that probably the tryptophan side chain can flip outwards away from the active site, thus making the pocket more readily accessible for the larger PI ligands. These data also suggest that both the position and specific combination of mutations are important for specificity towards PI substrates. The new acquired substrate specificity for PIs in the mutants comes with a loss in affinity for pTyr (Table 2), consistent with changes in the shape of the active site.

**Table 2:** Kinetics parameters of LMWPTPB-WT, mutants and MTMR2. All values are mean ± SEM of two experiments with triplicates. n.a: negligible or no detectable activity, n.d: not determined. pNPP assay was used for determination of kinetic parameters using pNPP as substrate; the malachite green assay was used for phosphor-tyrosine (pTyr) and phosphoinositides.

	WT			RT			RTK			RK			RKK			MTMR2		
Substrates	*K*_*m*_ (μM)	*k*_*cat*_ (s^-1^) x 10^−3^	*k*_*cat*_*/K*_*m*_ (M^-1^s^-1^) X 10^2^	*K*_*m*_ (μM)	*k*_*cat*_ (s^-1^) x 10^−3^	*k*_*cat*_*/K*_*m*_ (M^-1^s^-1^) X 10^2^	*K*_*m*_ (μM)	*k*_*cat*_ (s^-1^) x 10^−3^	*k*_*cat*_*/K*_*m*_ (M^-1^s^-1^) X 10^2^	*K*_*m*_ (μM)	*k*_*cat*_ (s^-1^) x 10^−3^	*k*_*cat*_*/K*_*m*_ (M^-1^s^-1^) X 10^2^	*K*_*m*_ (μM)	*k*_*cat*_ (s^-1^) x 10^−3^	*k*_*cat*_*/K*_*m*_ (M^-1^s^-1^) X 10^2^	*K*_*m*_ (μM)	*k*_*cat*_ (s^-1^) x 10^−3^	*k*_*cat*_*/K*_*m*_ (M^-1^s^-1^) X 10^2^
**pNPP**	476 ± 30.2	6033 ± 106	126.9 ± 6.7	655 ± 57	2559 ± 173	39.1 ± 4.4	1032 ± 26	1490 ± 57	14.4 ± 0.7	1388 ± 105	835 ± 118	6.0 ± 0.7	1717 ± 87	880 ± 126	5.1 ± 0.7	n.d	n.d	n.d
**pTyr**	307 ± 29	0.226 ± 0.019	0.735 ± 0.054	> 2000	n.d.	n.d.	> 2000	n.d.	n.d.	598 ± 232	0.025 ± 0.004	0.045 ± 0.021	> 2000	n.d.	n.d.	n.d.	n.d.	n.d.
**PI(3)P**	n.d	n.d	n.d	n.a	n.a	n.a	n.a	n.a	n.a	22.9 ± 5	3.9 ± 0.1	1.7 ± 0.2	n.a	n.a	n.a	139 ± 10	573 ± 7	41.5 ± 1.2
**PI(4)P**	n.d	n.d	n.d	n.a	n.a	n.a	n.a	n.a	n.a	30.7 ± 3.5	8.4 ± 1.8	2.7 ± 0.1	n.a	n.a	n.a	n.d	n.d	n.d
**PI(5)P**	n.d	n.d	n.d	29.2 ± 3.7	5.2 ± 0.2	1.8 ± 0.1	26.2 ± 3.9	3.7 ± 0.1	1.4 ± 0.1	35.9 ± 6.3	10.6 ± 1.1	3.0 ± 0.4	27.2 ± 7.0	6.5 ± 0.2	2.4 ± 0.03	n.d	n.d	n.d
**PI(3,4)P_2_**	n.d	n.d	n.d	n.a	n.a	n.a	n.a	n.a	n.a	n.a	n.a	n.a	n.a	n.a	n.a	n.d	n.d	n.d
**PI(3,5)P_2_**	n.d	n.d	n.d	n.a	n.a	n.a	n.a	n.a	n.a	21.84 ± 2.5	11.2 ± 0.5	5.11 ± 0.1	200.3 ± 42.2	26.7 ± 3.0	1.3 ± 0.1	62 ± 4	348 ± 17	57.3 ± 7
**PI(4,5)P_2_**	n.d	n.d	n.d	n.a	n.a	n.a	n.a	n.a	n.a	n.a	n.a	n.a	n.a	n.a	n.a	n.d	n.d	n.d
**PI(3,4,5)P_3_**	n.d	n.d	n.d	n.a	n.a	n.a	n.a	n.a	n.a	n.a	n.a	n.a	n.a	n.a	n.a	n.d	n.d	n.d

Interestingly, the RK mutant shows much higher affinity for PI3P than the phosphoinositide phosphatase MTMR2 (one order of magnitude) and also higher affinity for PI[3,5]P_2_ (3 times). In contrast, the catalytic efficiency (*k*_*cat*_*/K*_*m*_) is about one order on magnitude lower for both substrates, compared to MTMR2, due to the much lower turnover rate (*k*_*cat*_) (Table 2). Despite these differences, our Rosetta designs are remarkably more efficient than previously reported examples of computational enzyme redesign, where catalytic efficiencies are 9–11 orders of magnitude lower than the natural enzymes [48].

### Mutations do not disrupt the structural integrity of the enzyme

**Fig 3:**
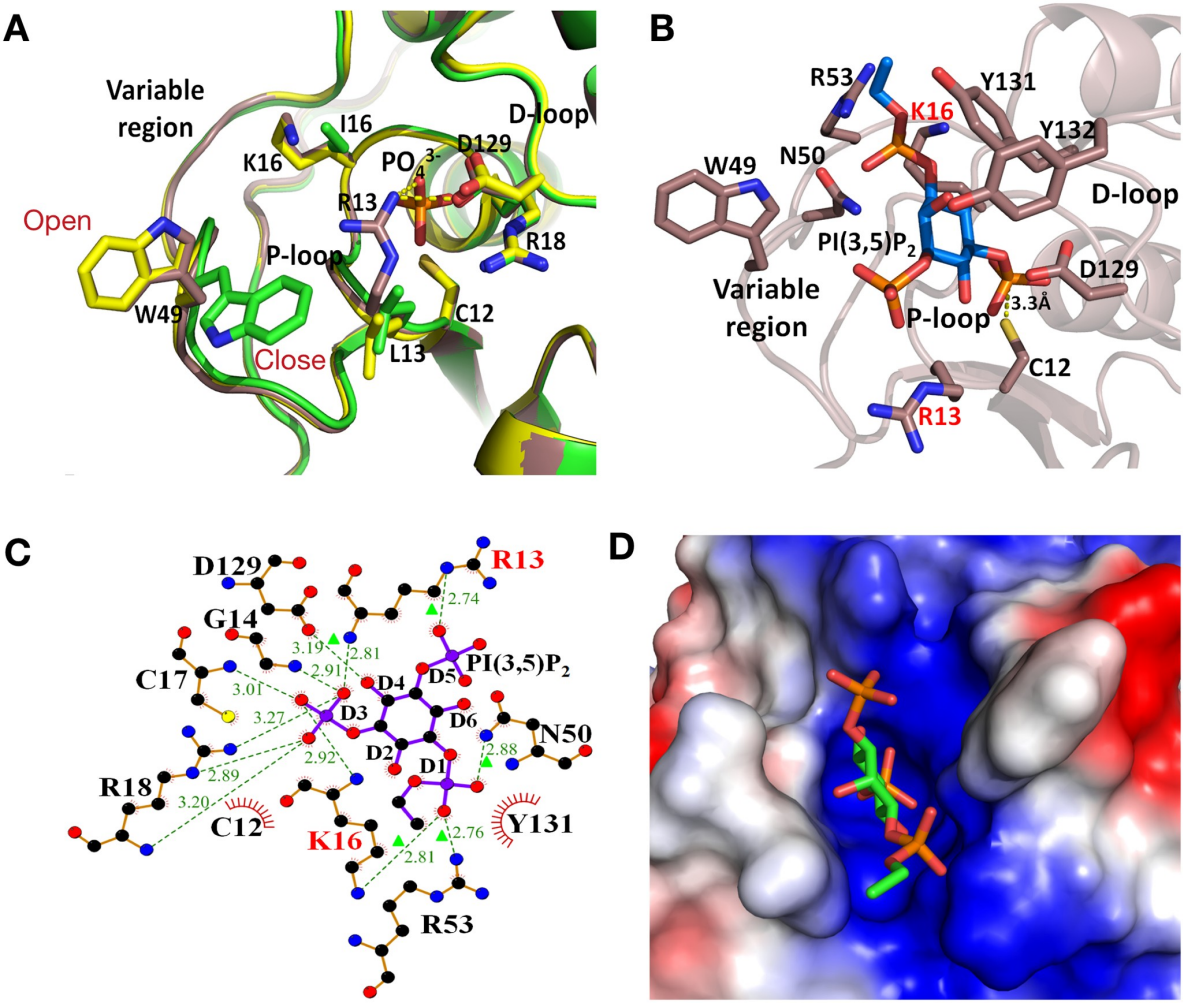
Crystallographic analysis of designed mutants. **A**) Superimposition of the crystal structures of LMW-PTP WT (green), K16 (yellow), R13-K16 (brown). A phosphate binds to active site of K16 and R13-K16 structures close to the catalytic residues (C12, R18 and D129) and it interacts with R13. **B**) Docking of PI[3,5]P_2_ ligand in the structure of R13-K16 (brown), showing the proximity of the D3 phosphate to the catalytic C12. **C**) Interactions of PI[3,5]P_2_ at the active site of R13-K16 after molecular docking calculated with Ligplot [47]. **D**) Electrostatic surface representation of the active site of the R13-K16 structure with PI[3,5]P_2_ docked in sticks.

In order to assess the actual impact of the designed mutations on the protein structure, we determined the crystal structures of the I16K and L13R-I16K mutants at 1.8Å and 2.0Å resolution respectively (Table 3). In both cases, the structures are similar to the WT enzyme with overall root-mean deviations (RMSDs) of 0.288 Å and 0.265 Å respectively. There are no structural distortions in main chain of the P-loop or the variable region (Fig 3A), thus the mutations introduced do not alter the tertiary structure of the protein, consistent with a catalytically active enzyme. However, in both structures, the side chain of W49 is in the *open* position, away from the active site (Fig 3A), thus confirming the flexibility of this side chain as suggested from the kinetics characterisation. In the structure of the RK mutant, the side chain of R13 points towards the centre of the active site interacting with a bound phosphate molecule and the catalytic D129 (Fig 3A).

**Table 3:** Crystallographic and refinement parameters of the L13R-I16K and I16K structures.

LMW-PTP mutants	L13R-I16K	I16K
Wavelength	0.98	0.98
Resolution range	33.36–2.0 (2.072–2.0)	30.87–1.769 (1.832–1.769)
Space group	C 1 2 1	C 1 2 1
Unit cell	56.625 41.287 56.898 90 91.809 90	57.152 41.76 62.15 90 96.573 90
Total reflections	29584 (3055)	44768 (4559)
Unique reflections	9025 (917)	13623 (1385)
Multiplicity	3.3 (3.3)	3.3 (3.3)
Completeness (%)	99.04 (99.78)	94.05 (99.50)
Mean I/sigma(I)	9.87 (2.02)	18.72 (2.16)
Wilson B-factor	24.41	28.03
R-merge	0.09589 (0.5859)	0.04712 (0.509)
R-meas	0.1148 (0.6991)	0.05623 (0.6092)
R-pim	0.06239 (0.3776)	0.03039 (0.3309)
CC1/2	0.995 (0.718)	0.997 (0.765)
CC*	0.999 (0.914)	0.999 (0.931)
Reflections used in refinement	8946 (917)	13491 (1383)
Reflections used for R-free	477 (46)	674 (86)
R-work	0.1823 (0.2421)	0.1633 (0.2651)
R-free	0.2312 (0.2706)	0.1962 (0.2593)
CC(work)	0.959 (0.855)	0.965 (0.878)
CC(free)	0.908 (0.645)	0.960 (0.867)
Number of non-hydrogen atoms	1343	1420
macromolecules	1237	1276
ligands	15	4
solvent	91	140
Protein residues	153	153
RMS(bonds)	0.002	0.01
RMS(angles)	0.47	1.18
Ramachandran favoured (%)	98.01	98.01
Ramachandran allowed (%)	2.65	1.99
Ramachandran outliers (%)	0	0
Rotamer outliers (%)	0	0
Clashscore	0.81	3.94
Average B-factor	30.64	33.93
macromolecules	30.12	32.88
ligands	44.06	32.48
solvent	35.41	43.54
Number of TLS groups	1	1

To evaluate if a PI molecule could fit in the crystal structure of the RK mutant, we performed docking with *RosettaLigand* using the PI[3,5]P_2_ ligand, and allowing the side chains of the protein residues in the binding interface (R13, I16, W49, N50, R53, Y131 and Y132) to be flexible. The ligand docks in the pocket, with the phosphorus atom of the D3 phosphate at a distance of 3.3Å from the sulphur atom of C12 (Fig 3B), similar to the 3.2Å distance observed in the MTMR2-PI[3,5]P_2_ complex [4]. The new residues R13 and K16, both form interactions with the D5 and D1 phosphates respectively, and together with interactions with R53 they contribute to PI[3,5]P_2_ binding (Fig 3C). The crystal structures and docking thus confirm that the introduction of two new basic residues does not disrupt the three-dimensional structure of the enzyme, but it is actually critical for efficient binding of the PI[3,5]P_2_ ligand and creates a more positively charged active site (Fig 3D) similar to that of MTMR2 (Fig 3B). Furthermore, the overall volume of the active site for the RK mutant (apo-enzyme from the docking simulation) is 861.5 Å^3^, larger than that of the WT enzyme of 546 Å^3^ (and of the RTKA and RKKA designs, see above). Thus a combination of larger pocket and the introduction of basic residues results in a suitable binding pocket to accommodate PI[3,5]P_2_, confirming our enzyme kinetics data.

## Discussion

In this study we have demonstrated that the specific activity of the LMW-PTP can be redesigned from p-Tyr specific to dephosphorylate up to four phosphoinositide substrates using a combination of rational and computational design with *RosettaEnzyme* [24]. The change in substrate specificity was achieved by mutating only two residues in the active site resulting in a larger active site and providing new interactions with the additional phosphatase groups in the PI substrates. Consistent with this, the mutant enzymes show a poor affinity for pTyr compared to the WT enzyme. Importantly, the crystal structures of the K16 and R13-K16 designs show that mutations introduced in the new enzymes do not alter the 3D structure of the protein or the position of the catalytic residues. This ensures that the catalytic mechanism remains unaltered in the mutants.

In total, we produced 11 new redesigned enzymes, 10 of which are active enzymes and able to dephosphorylate PI substrates, whereas the WT enzyme showed poor or no activity towards these substrates. The redesigned enzymes present a different range of substrate specificities, RK has broader specificity, RKK is specific for PI5P and PI[3,5P]_2_, while RT and RTK are selective for PI5P. The binding affinity for these mutants determined from the kinetics experiments is within the range of affinities observed in natural PI phosphatases (18–130 μM [4, 49, 50]), and in some instances even better.

The crystals structure of R13-K16 design shows a larger active site pocket than the WT enzyme, which is compatible with binding of PI substrates. Molecular docking highlights the similarities in the predicted binding mode of PI[3,5]P_2_ to the LMW-PTP-RK redesign compared to the natural receptor myotubularin. These similarities confirm our kinetics results and the high binding affinity observed. The newly introduced residues R13 and K16 create a more positively charged surface around the active site and are in a position to establish electrostatic interactions with the D1 and D5 phosphates in PI[3,5]P_2_, whereas N50 and R53 are also within hydrogen bond distance of the D1 oxygen atoms. The structure also explains why the designed enzymes did not show any detectable activity on PI[3,4,5]P_3_. The extra phosphate at position 4 of the inositol ring would clash with the residue at position 16 in a way that would make impossible to accommodate both D3 and D4 in the active site. However, D3 and D5 have the optimal spacing to bind to the P-loop and still bind to the additional residues in the variable region (N50 and R53) to stabilise binding.

Our results set the stage to pursue the redesign of LMW-PTP to function on a variety of different substrates. We have demonstrated in this study that it is a robust enzyme amenable to mutagenesis without disrupting its structure or catalytic activity. The results presented here thus offer an opportunity to investigate in the future the potential use of redesigned phosphatases for applications in human disease therapy, particularly in diseases resulting from direct dysfunction of PTPs. As with other engineered enzymes, LMW-PTP redesigns could also have alternative applications as research tools to investigate the regulation of signalling pathways.

## Supporting information

S1 Table Mutations introduced during Rosetta computational design and Rosetta binding energies for design strategy 1. LMWPTP-WT was used as a template and PI(3,5)P_2_ as ligand. Rosetta Energy Unit (REU) is an arbitrary unit for the Rosetta Binding Energy (RBE).
(DOCX)

S2 Table Mutations introduced during Rosetta computational design and Rosetta binding energies for design strategy 2. LMWPTP-I16K was used as template and PI(3,5)P_2_ as ligand. Rosetta Energy Unit (REU) is an arbitrary unit for the Rosetta Binding Energy (RBE).
(DOCX)
