# Supplementary material for: Computational and structure-guided design of phosphoinositide substrate specificity into the tyrosine specific LMW-PTP enzyme
Source: PLoS One. 2020 Jun 25;15(6):e0235133. doi: 10.1371/journal.pone.0235133 (PMC7316235; doi:10.1371/journal.pone.0235133)
Supplement: S1 Table — LMWPTP-WT was used as a template and PI(3,5)P2 as ligand. Rosetta Energy Unit (REU) is an arbitrary unit for the Rosetta Binding Energy (RBE). (DOCX) [file pone.0235133.s001.docx]

**Table S1. Mutations introduced during Rosetta computational design and Rosetta binding energies for design strategy 1.** LMWPTP-WT was used as a template and PI(3,5)P_2_ as ligand. Rosetta Energy Unit (REU) is an arbitrary unit for the Rosetta Binding Energy (RBE).

| **LMWPTPB-WT** | **LEU13** | **ILE16** | **TRP49** | **ASN50** | **TYR131** | **TYR132** | **RBE (REU)** |
| --- | --- | --- | --- | --- | --- | --- | --- |
| DE_1948 | ARG | THR | LYS | ASN | ALA | TYR | -10.14 |
| DE_741 | ARG | THR | LYS | ASN | ALA | TYR | -9.84 |
| DE_251 | ARG | THR | LYS | ASN | ALA | TYR | -9.83 |
| DE_837 | SER | THR | LYS | ASN | ALA | TYR | -9.52 |
| DE_1269 | SER | ALA | LYS | ASN | ALA | TYR | -9.44 |
| DE_1329 | SER | ASN | LYS | SER | TYR | TYR | -9.33 |
| DE_735 | SER | THR | ARG | ASN | ALA | TYR | -9.14 |
| DE_1125 | SER | THR | LYS | ASN | ALA | TYR | -9.11 |
| DE_88 | ARG | THR | LYS | ASN | TYR | TYR | -9.11 |
| DE_873 | ARG | THR | SER | ASN | ALA | TYR | -8.99 |
| DE_1080 | ARG | THR | ASN | ASN | ALA | TYR | -8.95 |
| DE_1541 | ARG | THR | ASN | ASN | ALA | TYR | -8.95 |
| DE_122 | SER | THR | LYS | ASN | TYR | TYR | -8.92 |
| DE_1299 | SER | THR | LYS | ASN | ALA | TYR | -8.9 |
| DE_359 | SER | THR | LYS | ASN | ALA | TYR | -8.8 |
| DE_13 | ARG | THR | LYS | ASN | ALA | TYR | -8.78 |
| DE_612 | SER | THR | LYS | ASN | ALA | TYR | -8.77 |
| DE_369 | ARG | THR | ARG | ASN | SER | TYR | -8.76 |
| DE_1833 | ARG | THR | ASN | ASN | ALA | TYR | -8.67 |
| DE_1883 | ARG | THR | LYS | ASN | ALA | TYR | -8.67 |
