# Supplementary material for: Computational and structure-guided design of phosphoinositide substrate specificity into the tyrosine specific LMW-PTP enzyme
Source: PLoS One. 2020 Jun 25;15(6):e0235133. doi: 10.1371/journal.pone.0235133 (PMC7316235; doi:10.1371/journal.pone.0235133)
Supplement: S2 Table — LMWPTP-I16K was used as template and PI(3,5)P2 as ligand. Rosetta Energy Unit (REU) is an arbitrary unit for the Rosetta Binding Energy (RBE). (DOCX) [file pone.0235133.s002.docx]

**Table S2. Mutations introduced during Rosetta computational design and Rosetta binding energies for design strategy 2.** LMWPTP-I16K was used as template and PI(3,5)P_2_ as ligand. Rosetta Energy Unit (REU) is an arbitrary unit for the Rosetta Binding Energy (RBE).

| **LMWPTPB-I16K** | **LEU13** | **LYS16*** | **TRP49** | **ASN50** | **TYR131** | **TYR132** | **RBE (REU)** |
| --- | --- | --- | --- | --- | --- | --- | --- |
| DE_316 | ARG | LYS | LYS | ASN | ALA | TYR | -9.17 |
| DE_1376 | ARG | LYS | LYS | ASN | ALA | TYR | -8.94 |
| DE_245 | ARG | LYS | LYS | ASN | ALA | TYR | -8.62 |
| DE_791 | ARG | LYS | LYS | ASN | ALA | TYR | -8.49 |
| DE_1299 | SER | LYS | ARG | ASN | SER | TYR | -8.38 |
| DE_408 | SER | LYS | LYS | ASN | ALA | TYR | -8.29 |
| DE_390 | ARG | LYS | SER | ASN | ALA | TYR | -7.93 |
| DE_755 | SER | LYS | LYS | ASN | ALA | TYR | -7.62 |
| DE_1818 | ARG | LYS | ASN | ASN | ALA | TYR | -7.6 |
| DE_1160 | ARG | LYS | ARG | ASN | SER | TYR | -7.49 |
| DE_1654 | SER | LYS | LYS | ASN | ALA | TYR | -7.48 |
| DE_165 | LEU | LYS | ARG | ASN | SER | TYR | -7.3 |
| DE_1108 | ARG | LYS | SER | ASN | ALA | TYR | -7.29 |
| DE_1036 | SER | LYS | LYS | ASN | ALA | TYR | -7.26 |
| DE_917 | SER | LYS | LYS | ASN | ALA | TYR | -7.25 |
| DE_1383 | SER | LYS | LYS | ASN | ALA | TYR | -7.23 |
| DE_1319 | SER | LYS | ASN | ASN | ALA | TYR | -7.21 |
| DE_291 | SER | LYS | LYS | ASN | ALA | TYR | -7.13 |
| DE_344 | ARG | LYS | SER | ASN | SER | TYR | -7.11 |
| DE_134 | SER | LYS | LYS | ASN | ALA | TYR | -7.06 |

*mutation was pre-set in the template model used for redesign
